# Supplementary material for: Ecological and social pressures interfere with homeostatic sleep regulation in the wild
Source: eLife. 2022 Mar 1;11:e73695. doi: 10.7554/eLife.73695 (PMC8887896; doi:10.7554/eLife.73695)
Supplement: Supplementary file 1. — (a) Individual metadata. Table depicts the sex, age, weight, capture date, as well as data collection start and end dates for each study individual. F, female; M, male; A, adult; SA, subadult; J, juvenile; ACC, accelerometry. (b) Pearson correlation coefficient between the metrics of sleep extracted from the accelerometry data. Total sleep time is correlated with all sleep metrics, and sleep fragmentation is correlated with sleep efficiency. (c) Model output table of model of total sleep time (for the first 20 days) with all numerical variables standardized. (d) Model output table of model of total sleep time (for the first 20 days) with no standardization of variables. (e) Model output table of model of total sleep time (for the first 20 days) with all numerical variables standardized (daytime vectorial dynamic body acceleration [VeDBA] included instead of travel distance). (f) Model output table of model of time spent napping during the day (for the first 20 days) with all numerical variables standardized. (g) Model output table of model of time spent napping during the day (for the first 20 days) without standardization of the variables. (h) Model output table of model of total sleep time using data from entire study duration (including after the leopard attack) with all variables standardized. (i) Model output table of model of total sleep time using data from entire study duration (including after the leopard attack) without standardization of variables. (j) Model output table of model of sleep fragmentation (for the first 20 days) with all numerical variables standardized. (k) Model output table of model of sleep fragmentation (for the first 20 days) with no standardization of variables. (l) Model output table of model of sleep fragmentation (for the first 20 days) with all numerical variables standardized (daytime VeDBA included instead of travel distance). (m) Model output table of model of sleep fragmentation using data from entire study duration (including [file elife-73695-supp1.docx]

**Supplementary File 1**

| Collar # | Sex | Age | Weight (kg) | Capture date | GPS/ACC start date | GPS end date | ACC end date |
| --- | --- | --- | --- | --- | --- | --- | --- |
| 2460 | F | A | 12 | 2012-07-28 | 2012-08-01 | 2012-08-01 | 2012-08-01 |
| 2459 | F | SA | 8.6 | 2012-07-22 | 2012-08-01 | 2012-08-01 | 2012-08-01 |
| 2458 | F | A | 13 | 2012-07-22 | 2012-08-01 | 2012-08-01 | 2012-08-01 |
| 2457 | M | A | 28 | 2012-07-21 | 2012-08-01 | 2012-09-04 | 2012-09-04 |
| 2456 | F | A | 17.5 | 2012-07-22 | 2012-08-01 | 2012-08-31 | 2012-08-31 |
| 2455 | F | SA | 10 | 2012-07-28 | 2012-08-01 | 2012-08-08 | 2012-08-08 |
| 2454 | M | J | 9.25 | 2012-07-22 | 2012-08-01 | 2012-08-14 | 2012-08-14 |
| 2453 | F | A | 11.2 | 2012-07-25 | 2012-08-01 | 2012-08-03 | 2012-08-03 |
| 2452 | M | SA | 9 | 2012-07-21 | 2012-08-01 | 2012-08-14 | 2012-08-14 |
| 2451 | F | A | 13 | 2012-07-22 | 2012-08-01 | 2012-09-02 | 2012-09-02 |
| 2450 | F | SA | 8.85 | 2012-07-24 | 2012-08-01 | 2012-08-05 | 2012-08-05 |
| 2449 | F | A | 12.75 | 2012-07-23 | 2012-08-01 | 2012-08-31 | 2012-08-31 |
| 2448 | M | J | 6.65 | 2012-07-22 | 2012-08-01 | 2012-08-16 | 2012-08-17 |
| 2447 | F | A | 12.25 | 2012-07-24 | 2012-08-01 | 2012-08-31 | 2012-08-31 |
| 2446 | F | A | 15.75 | 2012-07-25 | 2012-08-01 | 2012-09-02 | 2012-09-02 |
| 2443 | M | SA | 9.25 | 2012-07-23 | 2012-08-01 | 2012-09-02 | 2012-09-02 |
| 2441 | F | SA | 8 | 2012-07-22 | 2012-08-01 | 2012-08-29 | 2012-08-29 |
| 2439 | F | A | 16 | 2012-07-21 | 2012-08-01 | 2012-09-04 | 2012-09-04 |
| 2436 | M | SA | 16 | 2012-07-22 | 2012-08-01 | 2012-09-02 | 2012-09-02 |
| 2434 | M | A | 25 | 2012-07-22 | 2012-08-01 | 2012-08-02 | 2012-08-02 |
| 2433 | M | SA | 10 | 2012-07-22 | 2012-08-01 | 2012-08-06 | 2012-08-06 |
| 2432 | M | SA | 8 | 2012-07-22 | 2012-08-01 | 2012-08-05 | 2012-08-05 |
| 2430 | F | A | 14.65 | 2012-07-28 | 2012-08-01 | 2012-08-03 | 2012-08-03 |
| 2428 | F | SA | 8.5 | 2012-07-29 | 2012-08-01 | 2012-08-15 | 2012-08-15 |
| 2427 | M | A | 25 | 2012-07-21 | 2012-08-01 | 2012-09-04 | 2012-09-04 |
| 2426 | M | SA | 20 | 2012-07-21 | 2012-08-01 | 2012-08-30 | 2012-08-31 |

**Supplementary file 1a**. Individual metadata. Table depicts the sex, age, weight, capture date, as well as data collection start and end dates for each study individual. F = female, M = male, A = adult, SA = subadult, J = juvenile, ACC = accelerometry.

|  | Total sleep time | Sleep onset time | Waking time | Sleep period duration | Sleep efficiency | Sleep fragmentation |
| --- | --- | --- | --- | --- | --- | --- |
| Total sleep time | 1 | -0.61 | 0.65 | 0.87 | 0.57 | -0.48 |
| Sleep onset time | x | 1 | -0.04 | -0.72 | -0.05 | 0.03 |
| Waking time | x | x | 1 | 0.72 | 0.10 | -0.10 |
| Sleep period duration | x | x | x | 1 | 0.11 | -0.09 |
| Sleep efficiency | x | x | x | x | 1 | -0.83 |
| Sleep Fragmentation | x | x | x | x | x | 1 |

**Supplementary file 1b.** Pearson correlation coefficient between the metrics of sleep extracted from the accelerometry data. Total sleep time is correlated with all sleep metrics.

|  | Total sleep time (standardized) | |
| --- | --- | --- |
| *Predictors* | *Estimates* | *CI (95%)* |
| Intercept | 0.02 | -0.50 – 0.53 |
| Travel distance | -0.03 | -0.19 – 0.14 |
| Time spent napping | -0.19 | -0.36 – -0.01 |
| Previous night relative total sleep time | -0.04 | -0.19 – 0.12 |
| Previous night relative sleep fragmentation | -0.06 | -0.19 – 0.06 |
| Tree fidelity score | 0.21 | 0.05 – 0.36 |
| Relative number of baboons in tree | -0.53 | -0.87 – -0.19 |
| Minimum ambient temperature | -0.04 | -0.20 – 0.13 |
| Moon phase | 0.05 | -0.12 – 0.22 |
| age: Juvenile | -0.34 | -1.15 – 0.50 |
| age: Subadult | -0.34 | -0.81 – 0.14 |
| sex: Male | 0.44 | -0.05 – 0.94 |
| tree: tree2 | -0.26 | -1.08 – 0.55 |
| tree: tree3 | -0.19 | -1.06 – 0.66 |
| tree: tree4 | -0.85 | -1.35 – -0.33 |
| tree: tree5 | -0.27 | -0.79 – 0.24 |
| tree: tree6 | -0.02 | -0.66 – 0.63 |
| tree: tree7 | 0.05 | -0.68 – 0.79 |
| tree: tree8 | -0.28 | -0.77 – 0.20 |
| tree: tree10 | -0.29 | -1.04 – 0.47 |
| tree: tree11 | -0.26 | -0.84 – 0.30 |
| Random Effects | | |
| σ^2^ | 0.53 | |
| τ_00_ _night_ | 0.02 | |
| τ_00_ _tag_ | 0.09 | |
| ICC | 0.17 | |
| N _tag_ | 18 | |
| N _night_ | 18 | |
| Observations | 170 | |
| Marginal R^2^ / Conditional R^2^ | 0.345 / 0.419 | |

**Supplementary file 1c.** Model output table of model of total sleep time (for the first 20 days) with all numerical variables standardized

|  | Total sleep time (hours) | |
| --- | --- | --- |
| *Predictors* | *Estimates* | *CI (95%)* |
| Intercept | 19.09 | -24.89 – 63.56 |
| Travel distance (km) | -0.02 | -0.12 – 0.09 |
| Time spent napping (mins) | -0.01 | -0.01 – -0.00 |
| Previous night relative total sleep time (mins) | -0.00 | -0.00 – 0.00 |
| Previous night relative sleep fragmentation (bouts/hour of sleep) | -0.11 | -0.33 – 0.11 |
| Tree fidelity score | 0.61 | 0.16 – 1.08 |
| Relative number of baboons in tree | -1.61 | -2.67 – -0.52 |
| Minimum ambient temperature (degree Celsius) | -0.03 | -0.19 – 0.12 |
| Moon phase | 0.11 | -0.24 – 0.50 |
| age: Juvenile | -0.27 | -0.88 – 0.36 |
| age: Subadult | -0.27 | -0.63 – 0.08 |
| sex: Male | 0.34 | -0.02 – 0.72 |
| tree: tree2 | -0.20 | -0.82 – 0.45 |
| tree: tree3 | -0.14 | -0.81 – 0.52 |
| tree: tree4 | -0.66 | -1.04 – -0.26 |
| tree: tree5 | -0.22 | -0.61 – 0.18 |
| tree: tree6 | -0.01 | -0.50 – 0.49 |
| tree: tree7 | 0.05 | -0.52 – 0.63 |
| tree: tree8 | -0.23 | -0.60 – 0.15 |
| tree: tree10 | -0.21 | -0.80 – 0.37 |
| tree: tree11 | -0.21 | -0.66 – 0.22 |
| Random Effects | | |
| σ^2^ | 0.31 | |
| τ_00_ _night_ | 0.01 | |
| τ_00_ _tag_ | 0.05 | |
| ICC | 0.17 | |
| N _tag_ | 18 | |
| N _night_ | 18 | |
| Observations | 170 | |
| Marginal R^2^ / Conditional R^2^ | 0.342 / 0.417 | |

**Supplementary file 1d.** Model output table of model of total sleep time (for the first 20 days) with no standardization of variables

|  | Total sleep time (standardized) | |
| --- | --- | --- |
| *Predictors* | *Estimates* | *CI (95%)* |
| Intercept | 0.01 | -0.51 – 0.51 |
| Average VeDBA during day | -0.12 | -0.34 – 0.12 |
| Time spent napping | -0.13 | -0.31 – 0.05 |
| Previous night relative total sleep time | -0.04 | -0.18 – 0.11 |
| Previous night relative sleep fragmentation | -0.08 | -0.20 – 0.05 |
| Tree fidelity score | 0.21 | 0.05 – 0.36 |
| Relative number of baboons in tree | -0.57 | -0.91 – -0.21 |
| Minimum ambient temperature | -0.04 | -0.21 – 0.12 |
| Moon phase | 0.07 | -0.09 – 0.24 |
| age: Juvenile | -0.23 | -1.03 – 0.55 |
| age: Subadult | -0.27 | -0.78 – 0.18 |
| sex: Male | 0.53 | 0.08 – 0.97 |
| tree: tree2 | -0.33 | -1.15 – 0.49 |
| tree: tree3 | -0.51 | -1.30 – 0.29 |
| tree: tree4 | -0.89 | -1.41 – -0.37 |
| tree: tree5 | -0.31 | -0.83 – 0.21 |
| tree: tree6 | 0.00 | -0.63 – 0.62 |
| tree: tree7 | 0.05 | -0.69 – 0.79 |
| tree: tree8 | -0.30 | -0.79 – 0.20 |
| tree: tree10 | -0.18 | -0.90 – 0.55 |
| tree: tree11 | -0.28 | -0.84 – 0.28 |
| Random Effects | | |
| σ^2^ | 0.55 | |
| τ_00_ _night_ | 0.02 | |
| τ_00_ _tag_ | 0.07 | |
| ICC | 0.13 | |
| N _tag_ | 18 | |
| N _night_ | 18 | |
| Observations | 178 | |
| Marginal R^2^ / Conditional R^2^ | 0.358 / 0.419 | |

**Supplementary file 1e**. Model output table of model of total sleep time (for the first 20 days) with all numerical variables standardized (daytime VeDBA included instead of travel distance)

|  | Time spent napping (standardized) | |
| --- | --- | --- |
| *Predictors* | *Estimates* | *CI (95%)* |
| Intercept | -0.05 | -0.44 – 0.33 |
| Prior night total sleep time | 0.03 | -0.10 – 0.17 |
| Prior night sleep fragmentation | 0.00 | -0.11 – 0.12 |
| Random Effects | | |
| σ^2^ | 0.42 | |
| τ_00_ _night_ | 0.21 | |
| τ_00_ _tag_ | 0.44 | |
| ICC | 0.61 | |
| N _tag_ | 20 | |
| N _night_ | 19 | |
| Observations | 202 | |
| Marginal R^2^ / Conditional R^2^ | 0.005 / 0.616 | |

**Supplementary file 1f.** Model output table of model of time spent napping during the day (for the first 20 days) with all numerical variables standardized

|  | Time spent napping (minutes) | |
| --- | --- | --- |
| *Predictors* | *Estimates* | *CI (95%)* |
| Intercept | 17.52 | -9.03 – 45.26 |
| Prior night total sleep time (hours) | 1.16 | -1.62 – 3.86 |
| Prior night sleep fragmentation (bouts/hour of sleep) | 0.12 | -2.87 – 3.21 |
| Random Effects | | |
| σ^2^ | 226.61 | |
| τ_00_ _night_ | 40.73 | |
| τ_00_ _tag_ | 78.33 | |
| ICC | 0.34 | |
| N _tag_ | 20 | |
| N _night_ | 19 | |
| Observations | 202 | |
| Marginal R^2^ / Conditional R^2^ | 0.003 / 0.536 | |

**Supplementary file 1g.** Model output table of model of time spent napping during the day (for the first 20 days) without standardization of the variables

|  | Total sleep time (standardized) | |
| --- | --- | --- |
| *Predictors* | *Estimates* | *CI (95%)* |
| Intercept | -0.25 | -0.60 – 0.09 |
| cond_night: night of leopard attack | -0.20 | -0.85 – 0.45 |
| cond_night: first night in new sleep site | -1.52 | -2.15 – -0.86 |
| cond_night: second night in new sleep site | -0.29 | -0.98 – 0.42 |
| cond_night: third night in new sleep site | 0.24 | -0.45 – 0.90 |
| cond_night: remainder of nights (in original sleep site) | 0.27 | -0.05 – 0.58 |
| age: Juvenile | -0.24 | -1.10 – 0.61 |
| age: Subadult | -0.35 | -0.86 – 0.16 |
| sex: Male | 0.75 | 0.22 – 1.30 |
| Travel distance | -0.06 | -0.18 – 0.07 |
| Time spent napping | -0.12 | -0.28 – 0.05 |
| Previous night relative total sleep time | 0.18 | 0.06 – 0.29 |
| Previous night relative sleep fragmentation | 0.06 | -0.06 – 0.19 |
| Minimum ambient temperature | -0.01 | -0.15 – 0.14 |
| Moon phase | 0.02 | -0.12 – 0.16 |
| Random Effects | | |
| σ^2^ | 0.78 | |
| τ_00_ _tag_ | 0.15 | |
| ICC | 0.16 | |
| N _tag_ | 20 | |
| Observations | 275 | |
| Marginal R^2^ / Conditional R^2^ | 0.253 / 0.318 | |

**Supplementary file 1h.** Model output table of model of total sleep time using data from entire study duration (including after the leopard attack) with all variables standardized

|  | Total sleep time (hours) | |
| --- | --- | --- |
| *Predictors* | *Estimates* | *CI (95%)* |
| Intercept | 10.50 | -42.03 – 61.10 |
| cond_night: night of leopard attack | -0.17 | -0.88 – 0.53 |
| cond_night: first night in new sleep site | -1.17 | -1.87 – -0.47 |
| cond_night: second night in new sleep site | -0.24 | -0.97 – 0.49 |
| cond_night: third night in new sleep site | 0.15 | -0.57 – 0.89 |
| cond_night: remainder of nights (in original sleep site) | 0.17 | -0.17 – 0.49 |
| age: Juvenile | -0.27 | -0.96 – 0.41 |
| age: Subadult | -0.31 | -0.72 – 0.08 |
| sex: Male | 0.59 | 0.19 – 1.01 |
| Travel distance (km) | -0.04 | -0.14 – 0.05 |
| Time spent napping (mins) | -0.00 | -0.01 – 0.00 |
| Previous night relative total sleep time (mins) | 0.00 | 0.00 – 0.01 |
| Previous night relative sleep fragmentation (bouts/hour of sleep) | 0.12 | -0.10 – 0.33 |
| Minimum ambient temperature (degrees Celsius) | -0.00 | -0.18 – 0.18 |
| Moon phase | 0.07 | -0.34 – 0.48 |
| Random Effects | | |
| σ^2^ | 0.43 | |
| τ_00_ _night_ | 0.05 | |
| τ_00_ _tag_ | 0.09 | |
| ICC | 0.25 | |
| N _tag_ | 20 | |
| N _night_ | 32 | |
| Observations | 275 | |
| Marginal R^2^ / Conditional R^2^ | 0.262 / 0.369 | |

**Supplementary file 1i.** Model output table of model of total sleep time using data from entire study duration (including after the leopard attack) without standardization of variables

|  | Sleep fragmentation (standardized) | |
| --- | --- | --- |
| *Predictors* | *Estimates* | *CI (95%)* |
| Intercept | -0.27 | -0.93 – 0.37 |
| Travel distance | -0.10 | -0.31 – 0.11 |
| Time spent napping | 0.22 | 0.00 – 0.45 |
| Previous night relative total sleep time | 0.09 | -0.10 – 0.27 |
| Previous night relative sleep fragmentation | -0.03 | -0.18 – 0.12 |
| Tree fidelity score | -0.05 | -0.24 – 0.14 |
| Relative number of baboons in tree | 0.26 | -0.15 – 0.67 |
| Minimum ambient temperature | 0.04 | -0.17 – 0.26 |
| Moon phase | -0.08 | -0.30 – 0.13 |
| age: Juvenile | 0.72 | -0.28 – 1.75 |
| age: Subadult | 0.52 | -0.06 – 1.10 |
| sex: Male | -0.44 | -1.04 – 0.17 |
| tree: tree2 | 0.91 | -0.08 – 1.89 |
| tree: tree3 | 0.37 | -0.67 – 1.39 |
| tree: tree4 | 0.60 | -0.03 – 1.20 |
| tree: tree5 | 0.69 | 0.07 – 1.30 |
| tree: tree6 | 0.54 | -0.26 – 1.30 |
| tree: tree7 | -0.22 | -1.14 – 0.69 |
| tree: tree8 | -0.04 | -0.64 – 0.55 |
| tree: tree10 | -0.00 | -0.90 – 0.90 |
| tree: tree11 | 0.48 | -0.21 – 1.15 |
| Random Effects | | |
| σ^2^ | 0.79 | |
| τ_00_ _night_ | 0.04 | |
| τ_00_ _tag_ | 0.14 | |
| ICC | 0.19 | |
| N _tag_ | 18 | |
| N _night_ | 18 | |
| Observations | 170 | |
| Marginal R^2^ / Conditional R^2^ | 0.266 / 0.352 | |

**Supplementary file 1j.** Model output table of model of sleep fragmentation (for the first 20 days) with all numerical variables standardize

|  | Sleep fragmentation  (wake bouts / hour of sleep) | |
| --- | --- | --- |
| *Predictors* | *Estimates* | *CI (95%)* |
| Intercept | -5.74 | -44.05 – 31.71 |
| Travel distance (km) | -0.04 | -0.13 – 0.05 |
| Time spent napping (mins) | 0.01 | 0.00 – 0.01 |
| Previous night relative total sleep time (mins) | 0.00 | -0.00 – 0.00 |
| Previous night relative sleep fragmentation (bouts/hour of sleep) | -0.04 | -0.21 – 0.15 |
| Tree fidelity score | -0.10 | -0.47 – 0.26 |
| Relative number of baboons in tree | 0.55 | -0.33 – 1.45 |
| Minimum ambient temperature (degree Celsius) | 0.03 | -0.10 – 0.16 |
| Moon phase | -0.12 | -0.43 – 0.18 |
| age: Juvenile | 0.38 | -0.14 – 0.91 |
| age: Subadult | 0.28 | -0.02 – 0.57 |
| sex: Male | -0.23 | -0.55 – 0.08 |
| tree: tree2 | 0.50 | -0.03 – 1.01 |
| tree: tree3 | 0.22 | -0.34 – 0.77 |
| tree: tree4 | 0.33 | -0.00 – 0.66 |
| tree: tree5 | 0.37 | 0.04 – 0.72 |
| tree: tree6 | 0.29 | -0.12 – 0.71 |
| tree: tree7 | -0.10 | -0.58 – 0.40 |
| tree: tree8 | 0.00 | -0.31 – 0.32 |
| tree: tree10 | 0.02 | -0.47 – 0.51 |
| tree: tree11 | 0.27 | -0.10 – 0.64 |
| Random Effects | | |
| σ^2^ | -0.04 | |
| τ_00_ _night_ | 0.01 | |
| τ_00_ _tag_ | 0.00 | |
| ICC | -0.04 | |
| N _tag_ | -0.10 | |
| N _night_ | 0.55 | |
| Observations | 0.03 | |
| Marginal R^2^ / Conditional R^2^ | -0.12 | |

**Supplementary file 1k.** Model output table of model of sleep fragmentation (for the first 20 days) with no standardization of variables

|  | Sleep fragmentation (standardized) | |
| --- | --- | --- |
| *Predictors* | *Estimates* | *CI (95%)* |
| Intercept | -0.29 | -0.95 – 0.34 |
| Average VeDBA during day | -0.05 | -0.33 – 0.23 |
| Time spent napping | 0.20 | -0.02 – 0.42 |
| Previous night relative total sleep time | 0.07 | -0.11 – 0.25 |
| Previous night relative sleep fragmentation | -0.03 | -0.19 – 0.12 |
| Tree fidelity score | -0.06 | -0.25 – 0.13 |
| Relative number of baboons in tree | 0.24 | -0.18 – 0.65 |
| Minimum ambient temperature | 0.07 | -0.14 – 0.27 |
| Moon phase | -0.10 | -0.30 – 0.11 |
| age: Juvenile | 0.75 | -0.25 – 1.76 |
| age: Subadult | 0.62 | -0.00 – 1.24 |
| sex: Male | -0.46 | -1.04 – 0.12 |
| tree: tree2 | 0.85 | -0.13 – 1.82 |
| tree: tree3 | 0.45 | -0.51 – 1.40 |
| tree: tree4 | 0.55 | -0.07 – 1.17 |
| tree: tree5 | 0.65 | 0.03 – 1.28 |
| tree: tree6 | 0.43 | -0.34 – 1.22 |
| tree: tree7 | -0.21 | -1.08 – 0.70 |
| tree: tree8 | -0.05 | -0.64 – 0.56 |
| tree: tree10 | 0.09 | -0.79 – 0.97 |
| tree: tree11 | 0.38 | -0.28 – 1.06 |
| Random Effects | | |
| σ^2^ | 0.80 | |
| τ_00_ _night_ | 0.03 | |
| τ_00_ _tag_ | 0.14 | |
| ICC | 0.17 | |
| N _tag_ | 18 | |
| N _night_ | 18 | |
| Observations | 178 | |
| Marginal R^2^ / Conditional R^2^ | -0.29 | |

**Supplementary file 1l**. Model output table of model of sleep fragmentation (for the first 20 days) with all numerical variables standardized (daytime VeDBA included instead of travel distance)

|  | Sleep fragmentation (standardized) | |
| --- | --- | --- |
| *Predictors* | *Estimates* | *CI (95%)* |
| Intercept | 0.05 | -0.33 – 0.44 |
| cond_night: night of leopard attack | -0.32 | -0.96 – 0.33 |
| cond_night: first night in new sleep site | 0.96 | 0.33 – 1.60 |
| cond_night: second night in new sleep site | 0.10 | -0.60 – 0.80 |
| cond_night: third night in new sleep site | -0.48 | -1.16 – 0.20 |
| cond_night: remainder of nights (in original sleep site) | -0.20 | -0.52 – 0.11 |
| age: Juvenile | 0.69 | -0.26 – 1.60 |
| age: Subadult | 0.48 | -0.09 – 1.03 |
| sex: Male | -0.53 | -1.10 – 0.05 |
| Travel distance | 0.01 | -0.12 – 0.13 |
| Time spent napping | 0.21 | 0.05 – 0.38 |
| Previous night relative total sleep time | 0.02 | -0.10 – 0.14 |
| Previous night relative sleep fragmentation | -0.08 | -0.20 – 0.04 |
| Minimum ambient temperature | 0.10 | -0.04 – 0.24 |
| Moon phase | -0.05 | -0.18 – 0.09 |
| Random Effects | | |
| σ^2^ | 0.79 | |
| τ_00_ _tag_ | 0.20 | |
| ICC | 0.20 | |
| N _tag_ | 20 | |
| Observations | 275 | |
| Marginal R^2^ / Conditional R^2^ | 0.175 / 0.298 | |

**Supplementary file 1m.** Model output table of model of sleep fragmentation using data from entire study duration (including after the presumed leopard attack) with all variables standardized

|  | Sleep fragmentation  (wake bouts / hour of sleep) | |
| --- | --- | --- |
| *Predictors* | *Estimates* | *CI (95%)* |
| Intercept | -15.99 | -47.39 – 14.91 |
| cond_night: night of leopard attack | -0.16 | -0.58 – 0.24 |
| cond_night: first night in new sleep site | 0.50 | 0.07 – 0.92 |
| cond_night: second night in new sleep site | 0.06 | -0.38 – 0.51 |
| cond_night: third night in new sleep site | -0.24 | -0.67 – 0.19 |
| cond_night: remainder of nights (in original sleep site) | -0.11 | -0.31 – 0.10 |
| age: Juvenile | 0.37 | -0.11 – 0.85 |
| age: Subadult | 0.25 | -0.05 – 0.53 |
| sex: Male | -0.27 | -0.55 – 0.02 |
| Travel distance (km) | 0.00 | -0.06 – 0.06 |
| Time spent napping (mins) | 0.00 | 0.00 – 0.01 |
| Previous night relative total sleep time (mins) | 0.00 | -0.00 – 0.00 |
| Previous night relative sleep fragmentation (bouts/hour of sleep) | -0.09 | -0.23 – 0.05 |
| Minimum ambient temperature (degrees Celsius) | 0.06 | -0.05 – 0.17 |
| Moon phase | -0.06 | -0.30 – 0.19 |
| Random Effects | | |
| σ^2^ | 0.19 | |
| τ_00_ _night_ | 0.01 | |
| τ_00_ _tag_ | 0.05 | |
| ICC | 0.24 | |
| N _tag_ | 20 | |
| N _night_ | 32 | |
| Observations | 275 | |
| Marginal R^2^ / Conditional R^2^ | 0.185 / 0.329 | |

**Supplementary file 1n.** Model output table of model of sleep fragmentation using data from entire study duration (including after the presumed leopard attack) without standardization of variables

|  | Proportion of minutes synchronized (standardized) | |
| --- | --- | --- |
| *Predictors* | *Estimates* | *CI (95%)* |
| Intercept | -0.21 | -0.45 – 0.03 |
| Occupying same tree | 0.56 | 0.47 – 0.64 |
| Random Effects | | |
| σ^2^ | 0.60 | |
| τ_00_ _dy_name_ | 0.14 | |
| τ_00_ _night_ | 0.19 | |
| τ_00_ _tag1_ | 0.08 | |
| τ_00_ _tag2_ | 0.09 | |
| ICC | 0.45 | |
| N _night_ | 34 | |
| N _tag1_ | 22 | |
| N _tag2_ | 22 | |
| N _dy_name_ | 250 | |
| Observations | 2997 | |
| Marginal R^2^ / Conditional R^2^ | 0.050 / 0.404 | |

**Supplementary file 1o.** Model output table of model of synchronization (i.e. the proportion of minutes during a night that both dyad members exhibit the same behavior, either sleep or wakefulness) with response variable standardized of the response variable

|  | Proportion of minutes synchronized | |
| --- | --- | --- |
| *Predictors* | *Estimates* | *CI (95%)* |
| Intercept | 0.73 | 0.71 – 0.74 |
| Occupying same tree | 0.03 | 0.02 – 0.03 |
| Random Effects | | |
| σ^2^ | 0.00 | |
| τ_00_ _dy_name_ | 0.00 | |
| τ_00_ _night_ | 0.00 | |
| τ_00_ _tag1_ | 0.00 | |
| τ_00_ _tag2_ | 0.00 | |
| ICC | 0.45 | |
| N _night_ | 34 | |
| N _tag1_ | 22 | |
| N _tag2_ | 22 | |
| N _dy_name_ | 250 | |
| Observations | 2997 | |
| Marginal R^2^ / Conditional R^2^ | 0.050 / 0.404 | |

**Supplementary file 1p.** Model output table of model of synchronization (i.e. the proportion of minutes during a night that both dyad members exhibit the same behavior, either sleep or wakefulness) without standardization of the response variable

|  | Focal baboon awake in the current epoch | |
| --- | --- | --- |
| *Predictors* | *Odds Ratios* | *CI (95%)* |
| Intercept | 0.07 | 0.07 – 0.08 |
| At least one group-mate in the same sleep tree awake in previous epoch | 1.29 | 1.18 – 1.40 |
| Previous night relative total sleep time | 1.04 | 0.98 – 1.11 |
| Previous night relative sleep fragmentation | 1.01 | 0.96 – 1.06 |
| At least one neighbor awake x previous night relative total sleep time | 0.99 | 0.88 – 1.11 |
| At least one neighbor awake x previous night relative sleep fragmentation | 0.99 | 0.90 – 1.09 |
| Random Effects | | |
| σ^2^ | 3.29 | |
| τ_00_ _night_ | 0.01 | |
| τ_00_ _tag_ | 0.01 | |
| ICC | 0.00 | |
| N _night_ | 12 | |
| N _tag_ | 20 | |
| Observations | 44306 | |
| Marginal R^2^ / Conditional R^2^ | 0.001 / 0.002 | |

**Supplementary file 1q.** Model output table of model of an individual being awake in a given epoch. The previous night relative total sleep time and previous night relative sleep efficiency variables are standardized. Data that was modelled here was a subset of the full dataset, in which the focal baboon was not awake in the previous epoch, and the current epoch occurred between 21:00 and 05:00 and prior to the 15^th^ day of the study, when several of the baboons’ collars ceased collecting data.

*Sleep validation study*

To evaluate whether the accelerometer-based sleep classification technique was accurately monitoring sleep in baboons, we returned to Mpala Research Centre in July 2019 to perform a validation study in which we compared the results of the accelerometer-based sleep classification to direct observations of awake and sleeping baboons. Using the procedures described in Strandburg-Peshkin et al., 2015, we trapped and anesthetized 27 members of a group of habituated olive baboons, fitting each with a GPS and accelerometry collar. Eleven of the 27 collars deployed recorded continuous tri-axial accelerations at 12 Hz/axis from 06:30 to 18:00 and 0.71-second bursts of accelerations at 56.2 Hz/axis at the beginning of every minute from 18:00 to 06:30. Accelerometry data was collected by each of these 11 collars for up to 31 days. The remaining 16 collars did not collect accelerometry data from 06:30 to 18:00, and thus we excluded data from these collars from the validation study.

We down-sampled and interpolated the accelerometry data such that it matched the sampling frequency and schedule of the data collected in 2012 (i.e. the data analyzed for this manuscript). We then applied the sleep classification algorithm described in the Materials and Methods to this validation dataset.

To validate the sleep classification algorithm, we performed direct behavioral observations of the baboons at their primary sleep site. We recorded the behavior of the study baboons starting when they approached their sleep site using a FLIR T1020 high-resolution infrared camera (FLIR Systems Inc., Wilsonville, OR, USA). Recordings continued into the night for as long as the camera battery allowed (average recording duration (range of recording durations): 7.4 hours (1.7 – 14.9 hours)), and we collected thermal imaging data on 21 nights. We identified individuals in the thermal imagery both in real-time, via observer narration of the recorded imagery, and post-recording, by matching movements of individuals in the thermal imagery to the GPS tracks of collared individuals.

Following initial data collection, we used the commercial software Loopy (Loopbio GmbH, Austria) to score the behavior of identified individuals in the thermal imagery. Individuals’ behavior was scored as “wakefulness”, “resting wakefulness”, or “sleep” (Figure 5-figure supplement 1). Wakefulness refers to any behavior involving active movement (i.e. walking, running) or engaged activity (i.e. allogrooming), whereas resting wakefulness refers to behaviors that are dormant (i.e. sitting), but not in the typical sleeping posture of a baboon (sitting or lying with neck relaxed and head hung). Sustained dormant behavior in the typical sleep posture was considered sleep. Video scoring resulted in a total of 8.0 hours of behavioral observation across a total of 16 individual baboons.

Synchronizing the thermal imagery data with the accelerometry data produced a validation dataset of 301 minute-epochs across seven baboons that were both classified as either sleep or wakeful behavior from accelerometry, and scored as wakefulness, resting wakefulness, or sleep from direct observation. With both wakefulness and resting wakefulness representing wakeful behavior, the accelerometer-based sleep classification exhibited an accuracy of 80.7% (Supplementary file 1r). Consistent with previous validation studies of the use of accelerometry in measuring sleep (Ancoli-Israel et al., 2003; de Souza et al., 2003), we found that accelerometer-based sleep classification has difficulty distinguishing resting wakefulness from sleep, and we consider this limitation in our interpretation of the results.

|  | | **Behavioral scoring** | | |
| --- | --- | --- | --- | --- |
|  |  | Awake | | Asleep |
|  |  | Wakefulness | Resting wakefulness | Sleep |
| Accelerometer-based sleep classification | Awake | 32 | 89 | 17 |
|  | Asleep | 0 | 41 | 122 |

**Supplementary file 1r.** Confusion matrix reporting the results of the validation study. Table entries represent the number of minute-epochs classified according to the accelerometer-based technique and direct behavioral observation.
