# Supplementary material for: Ecological and social pressures interfere with homeostatic sleep regulation in the wild
Source: eLife. 2022 Mar 1;11:e73695. doi: 10.7554/eLife.73695 (PMC8887896; doi:10.7554/eLife.73695)
Supplement: Transparent reporting form [file elife-73695-transrepform1.pdf]

Westbrook Centre, Milton Road
Cambridge CB4 1YG
UK

P 01223 855340
W elifesciences.org
T @elife

eLife's transparent reporting form

We encourage authors to provide detailed information *within their submission* to facilitate the interpretation and replication of experiments. Authors can upload supporting documentation to indicate the use of appropriate reporting guidelines for health-related research (see EQUATOR Network), life science research (see the BioSharing Information Resource), or the ARRIVE guidelines for reporting work involving animal research. Where applicable, authors should refer to any relevant reporting standards documents in this form.

If you have any questions, please consult our Journal Policies and/or contact us: editorial@elifesciences.org.

Sample-size estimation

- You should state whether an appropriate sample size was computed when the study was being designed
- You should state the statistical method of sample size computation and any required assumptions
- If no explicit power analysis was used, you should describe how you decided what sample (replicate) size (number) to use

Please outline where this information can be found within the submission (e.g., sections or figure legends), or explain why this information doesn't apply to your submission:

Data collected represented the GPS and accelerometry tracking of 26 individuals during a 36-night study of sleep. All LMM and GAMM statistical procedures included individual identity and the night of data collection as random effects. More information can be found under the Data Collection section (L379-391). Elaboration on data collection per individual can be found in Supplementary file 1a

Replicates

- You should report how often each experiment was performed
- You should include a definition of biological versus technical replication
- The data obtained should be provided and sufficient information should be provided to indicate the number of independent biological and/or technical replicates
- If you encountered any outliers, you should describe how these were handled
- Criteria for exclusion/inclusion of data should be clearly stated
- High-throughput sequence data should be uploaded before submission, with a private link for reviewers provided (these are available from both GEO and ArrayExpress)

Please outline where this information can be found within the submission (e.g., sections or figure legends), or explain why this information doesn't apply to your submission:

eLife Sciences Publications, Ltd is a limited liability non-profit non-stock corporation incorporated in the State of Delaware, USA, with company number 5030732, and is registered in the UK with company number FC030576 and branch number BR015634 at the address Westbrook Centre, Milton Road Cambridge CB4 1YG, UK | March 2019

Data collected are observational and do not include experimental replicates. Accelerometry data were excluded when individuals were missing large portions of data from a particular data (specific criteria for exclusion can be found on L424-434). GPS data were excluded when individuals had incomplete days of data or collar malorientation (L451-459). All data that met inclusion criteria (or rather, that did not fall under the exclusion criteria) were included in statistical analyses.

Statistical reporting

- Statistical analysis methods should be described and justified
- Raw data should be presented in figures whenever informative to do so (typically when N per group is less than 10)
- For each experiment, you should identify the statistical tests used, exact values of N, definitions of center, methods of multiple test correction, and dispersion and precision measures (e.g., mean, median, SD, SEM, confidence intervals; and, for the major substantive results, a measure of effect size (e.g., Pearson's r, Cohen's d)
- Report exact p-values wherever possible alongside the summary statistics and 95% confidence intervals. These should be reported for all key questions and not only when the p-value is less than 0.05.

Please outline where this information can be found within the submission (e.g., sections or figure legends), or explain why this information doesn't apply to your submission:

Statistical analysis methods are described in L529-595.
For each finding, the statistical test used, exact p-value, effect size, and credible intervals, where applicable, are presented in the results section (L148-222).
Further details of each statistical analysis can be found in the Supplementary file 1.

(For large datasets, or papers with a very large number of statistical tests, you may upload a single table file with tests, Ns, etc., with reference to sections in the manuscript.)

Group allocation

- Indicate how samples were allocated into experimental groups (in the case of clinical studies, please specify allocation to treatment method); if randomization was used, please also state if restricted randomization was applied
- Indicate if masking was used during group allocation, data collection and/or data analysis

Please outline where this information can be found within the submission (e.g., sections or figure legends), or explain why this information doesn't apply to your submission:

Due to the observational nature of the work, no group allocation procedures were used

Additional data files ("source data")

- We encourage you to upload relevant additional data files, such as numerical data that are represented as a graph in a figure, or as a summary table
- Where provided, these should be in the most useful format, and they can be uploaded as “Source data” files linked to a main figure or table
- Include model definition files including the full list of parameters used
- Include code used for data analysis (e.g., R, MatLab)
- Avoid stating that data files are “available upon request”

Please indicate the figures or tables for which source data files have been provided:

GPS and accelerometry data generated during this study are published and available in the Movebank repository (www.movebank.org; Crofoot et al. 2021). Drone imagery is publicly available for download from Dryad (http://dx.doi.org/10.5061/dryad.6h5b7).

Accelerometry data and behavioral scoring data from the 2019 sleep validation study is also publicly available for download from Dryad (https://doi.org/10.5061/dryad.p5hqbzkqf).

All code used to produce the final results from the raw data is published in the repository CarterLoftus/baboon_sleep on GitHub, and the repository has been archived on Zenodo (Loftus & Harel 2022). Instructions on how to use the code to replicate the analyses are thoroughly detailed on the repository’s README.md file.

Crofoot MC, Kays RW, Wikelski M. 2021. Collective movement in wild baboons. Movebank Data Repository. https://doi.org/10.5441/001/1.3q2131q5

Loftus JC, Harel R. 2022. baboon_sleep. GitHub. https://github.com/CarterLoftus/baboon_sleep/. v1.0.0. https://doi.org/10.5281/zenodo.5905742
